# Use of DNA Barcodes to Identify Invasive Armyworm *Spodoptera* Species in Florida

**DOI:** 10.1673/031.011.15401

**Published:** 2011-11-10

**Authors:** Rodney N. Nagoshi, Julieta Brambila, Robert L. Meagher

**Affiliations:** ^1^ Center for Medical, Agricultural and Veterinary Entomology, USDA-ARS, Gainesville, FL 32608; ^2^ Entomology, USDA APHIS PPQ, Gainesville, FL 32604

**Keywords:** COI, cytochrome-c oxidase subunit I, invasive pests, mitochondrial DNA

## Abstract

A critical component for sustaining adequate food production is the protection of local agriculture from invasive pest insects. Essential to this goal is the ability to accurately distinguish foreign from closely related domestic species, a process that has traditionally required identification using diagnostic morphological “keys” that can be both subtle and labor-intensive. This is the case for the Lepidopteran group of insects represented by *Spodoptera*, a genus of Noctuidae “armyworm” moths that includes several important agricultural pests. Two of the most destructive species, *Spodoptera littoralis* (Boisduval) (Lepidoptera: Noctuidae) and *S. litura* (F.) are not yet established in North America. To facilitate the monitoring for these pests, the feasibility of using DNA barcoding methodology for distinguishing between domestic and foreign *Spodoptera* species was tested. A DNA barcoding database was derived for a subset of *Spodoptera* species native to Florida, with an emphasis on those attracted to pheromone blends developed for *S. litura* or *S. littoralis.* These were then compared to the barcode sequences of *S. litura* collected from Taiwan and *S. littoralis* from Portugal. Consistent discrimination of the different species was obtained with phenetic relationships produced that were generally in agreement with phylogenetic studies using morphological characteristics. The data presented here indicate that DNA barcoding has the potential to be an efficient and accurate supplement to morphological methods for the identification of invasive *Spodoptera* pests in North America.

## Introduction

The genus *Spodoptera* (Lepidoptera: Noctuidae) includes some of the most important pests of agricultural crops in the world. These are commonly known as “armyworms” because severe infestations can appear as large masses “marching” in search of food; as many as 30 species have been described with members present on six continents. At least nine *Spodoptera* species are native to Florida (Heppner 1998), including those of greatest economic importance to North American agriculture, i.e., the southern armyworm *S. eridania* (Stoll), the beet armyworm *S. exigua* (Hübner), and the fall armyworm *S. frugiperda* (J. E. Smith).

Because *Spodoptera* species are of tropical origin, their overwintering ranges are typically limited to areas with mild winters, though some can diapause and thereby survive more extreme conditions. However, many of the native species are capable of extensive migrations and thus can cause seasonal damage well outside their overwintering range. The North American distribution of fall armyworm for example is limited to southern Florida and Texas during the winter months, but infestations extend as far north as Canada during the summer and fall (Luginbill 1928). Therefore, the establishment of invasive and migratory *Spodoptera* species into Florida is of special concern, as these can serve as source populations for migratory infestations into the rest of the continental United States.

Posing the greatest invasive threat on the USDA-APHIS-PPQ quarantine list are the Egyptian cotton leafworm, *S.* *littoralis* (Boisduval), and the tobacco cutworm, *S. litura* (F.) (Ellis 2004). Both species are highly polyphagous and produce economically significant damage to a range of crops, most notably cotton, soybean, maize, rice, and ornamentals, and each has a wide geographical distribution. *Spodoptera littoralis* is found in southern Europe, Africa, and the Middle East. The *S. litura* range includes the Middle East, most of Asia, Australia, and extends into the south Pacific as far west as Hawaii (Ellis 2004; Pogue 2002). From 1985 to 2010 there have been 172 and 663 interceptions of *S. littoralis* and *S. litura* in USA ports, respectively, with another 2809 interceptions identified as of the *Spodoptera* genus but not further classified (USDA-APHIS-PPQ 2010). A 2004 report indicated that most *S.* *littoralis* were intercepted in permit cargo flowers from Israel, while the majority of *S. litura* was found in permit cargo orchids (Ellis 2004). Florida is among the largest producers and distributors of floriculture in the USA, and thus is particularly susceptible to invasion by these Old World *Spodoptera* species (USDA-National Agricultural Statistics Service 2008). It is predicted that both species have the potential to become established in the southwestern and southeastern USA, reaching as far north as Maryland, and with annual migratory potential extending into Canada (Ellis 2004).

The availability of synthetic pheromone blends that attract *S. litura* and *S. littoralis* make possible the use of pheromone trapping as an efficient means of monitoring for the early establishment of these species in vulnerable areas (Neumark et al. 1977; Nemoto et al. 1980). The potential of this method for Florida was tested in areas near orchid nurseries that receive many imported plants (Meagher et al. 2008). Out of almost 200 specimens captured in traps baited with an *S. litura* pheromone blend, one *S. litura* specimen was found, with the remainder identified by morphology as belonging to native species. These results indicate that while pheromone trapping can be effective for monitoring large areas for invasive *Spodoptera*, their limited specificity still requires screening a large number of specimens.

Unfortunately, *S. litura* and *S.* *littoralis* have similar morphology to many domestic *Spodoptera* species, often making their distinction by classical physical criteria difficult. The most diagnostic morphological keys require microscopic characterization of adult male genital structures, a tedious procedure when screening large numbers, and one that requires substantial sample preparation and undamaged specimens (Pogue 2002). Unambiguous keys are frequently not available for females or immature stages, and substantial overlap in host range and attraction to pheromone blends limit the use of behavioral criteria (reviewed in Pogue 2002; Meagher et al. 2008). Therefore, finding an alternative method to supplement morphometric analyses is of practical interest for the *Spodoptera* complex.

DNA barcoding has been proposed as a molecular method for assigning individual specimens to known species (Hebert et al. 2003). The barcode involves DNA sequence analysis of a portion (typically between 600–900 bp) of the mitochondrial gene cytochrome *c* oxidase subunit I (COI). The central assumption is that barcode variation between even closely related species will be substantially greater than that observed within species. This is known as the “barcoding gap”, with a 10-fold difference between mean interspecific and intraspecific variations being frequently mentioned as a minimum threshold for the unambiguous assignment of unknown individuals to a species (Hebert et al. 2004). While this remains a favored criterion of many barcode proponents, even the absence of such gaps may still allow accurate assignment of barcode sequences to species in at least some taxa (Lou and Golding 2010; Virgilio et al. 2010).

To date, barcoding has had mixed success for species assignment in Lepidoptera. There are several examples of inaccurate species assignments using DNA barcoding that identify potential limitations in the methodology (Elias et al. 2007; Dasmahapatra et al. 2010). These were associated with problems arising from incomplete barcode coverage of existing diversity, the apparent absence of consistent barcode gaps in certain taxa, and the potentially confounding effects of incomplete lineage sorting that can be difficult to assess (reviewed in Rubinoff et al. 2006; Silva-Brandao et al. 2009). However, the technique was successfully applied in a study where 150 lepidopteran specimens were correctly assigned using a barcode database of 200 closely allied species (Hebert et al. 2003). In another large survey, a barcode comparison of about 100,000 specimens representing approximately 3500 species of moths, butterflies, flies, and wasps produced no misidentifications when a full barcode was available (Janzen et al. 2009). Perhaps most relevant to this paper, barcode comparisons were able to distinguish between four closely related *Helicoverpa* species, a complex in the same family (Noctuidae) as *Spodoptera* (Behere et al. 2007). These observations indicate that while the successful application of barcoding for species assignment may be taxa-dependent, with poorly studied or recently diverging groups being particularly problematic, the method has potential for facilitating the identification of invasive pest arthropods (Armstrong and Ball 2005; Darling and Blum 2007; Floyd et al. 2010).

The accuracy of species assignment by barcode comparisons is in theory dependent upon there being sufficient sampling of the target population and closely related nontarget species to both assess the existence of a barcode gap and to confidently estimate phylogenetic relationships (see for example Wiemers and Fiedler 2007; Darling and Blum 2007). The establishment of representative barcode databases for the exotic population and relevant native species is a potentially major undertaking that could require hundreds of sequences depending upon the genetic variability and similarity of the populations being compared. Therefore, an empirical demonstration of the feasibility of using DNA barcoding for a given taxa and region is prudent.

The objective of this study was to assess the applicability of DNA barcoding to monitor invasive *Spodoptera* species in Florida. Reference barcode databases were developed for a subset of *Spodoptera* native to Florida known to be attracted to *S.* *litura* pheromone traps (Meagher et al. 2008) commonly found on host plants associated with *S.* *litura* and *S.* *littoralis* (reviewed in Pogue 2002), and/or are important agricultural pests in North America. These databases were compared to barcode sequences from *S.* *littoralis* and *S. litura* specimens collected from Portugal and Taiwan, respectively, as identified by morphological criteria. The results were assessed for the likelihood of barcode gaps sufficient to discriminate the native from the foreign populations and thereby justify the expansion of the barcode databases for these and other related species. The potential role of DNA barcoding in the monitoring for invasive *Spodoptera* in Florida is discussed.

## Materials and Methods

### Specimen collections and sites.

The identification, collection, and processing of adult and larval specimens of *S. frugiperda* were described in previous studies (Nagoshi et al. 2006; Nagoshi et al. 2007). Other *Spodoptera* specimens were adult males obtained from pheromone-based traps (Table 1) with captures from each location representing pooled collections from multiple local sites and times. Standard plastic Universal moth traps (Unitraps) were baited with the appropriate commercially available pheromone blends (Suterra LLC, www.suterra.com) and contained insecticide strips (Hercon Environmental, www.herconenviron.com). After collection, specimens were typically stored at -20 °C. The species identity was determined by examination of male genital structures (illustrated in Pogue 2002). Abdomens were dissected and soaked in 70% isopropyl alcohol for indefinite storage. To prepare for examination, the abdomens were cleared in 10% KOH and incubated at 70 °C in a water bath for 45 min until clearing by visual inspection. They were then rinsed twice with isopropyl alcohol and the genitalia cleaned under a dissecting microscope. Morphological examination was done under alcohol or as permanently mounted specimens.

### DNA preparation and amplification of the COI region.

**Figure 1.**
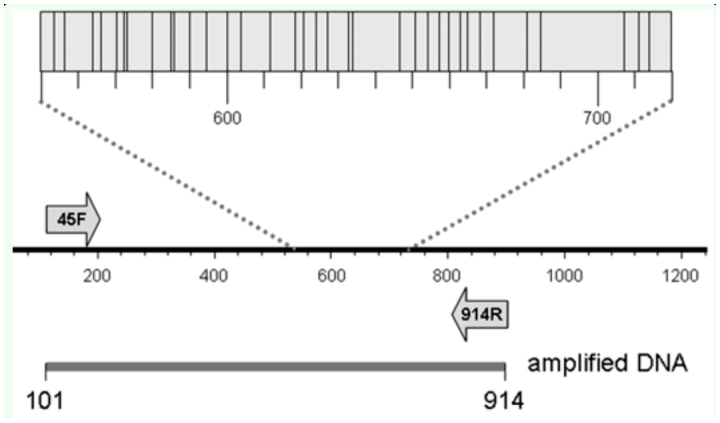
Diagram of the COI region used for barcoding. Arrows identify primers used for PCR amplification and DNA sequencing. Top box describes an approximately 200 bp region with the locations of species diagnostic nucleotide polymorphisms indicated by vertical lines (Table 4). High quality figures are available online.

Mitochondrial DNA was isolated from adults or larvae as previously described (Nagoshi et al. 2006). The DNA preparation was diluted to a final volume of 40 µl with distilled water. Genomic DNA preparations of fall armyworm samples from previous studies were stored at -20 °C (Table 1). PCR amplification was performed in a 30 µl reaction mix containing 3 µl 10X manufacturer's reaction buffer, 1 µl 10mM dNTP, 0.5 µl 20 µM primer mix, 1 µl DNA template (between 0.05–0.5 µg), 0.5 unit Taq DNA polymerase (New England Biolabs, www.neb.com). The thermocycling program was 94 °C (1 min), followed by 33 cycles of 92 °C (30 sec), 52 °C (45 sec), 72 °C (45 sec), and a final segment of 72 °C for 3 min. Amplification products were analyzed and isolated by agarose gel electrophoresis where 6 µl of 6X gel loading buffer was added to each amplification reaction, and the entire sample run on a 1.5% agarose horizontal gel containing GelRed (Biotium, www.biotium.com) in 0.5X Tris-borate buffer (TBE, 45 mM Tris base, 45 mM boric acid, 1 mM EDTA pH 8.0). A single band corresponding to the expected size of the amplified fragment was obtained from each reaction. To purify the amplified fragment away from excess primers, the fragment was visualized on a long-wave UV light box and cut out from the gel. Fragment isolation was performed using Zymo-Spin I columns (Zymo Research Corporation, www.zymoresearch.com) according to manufacturer's instructions. Primers were synthesized by Integrated DNA Technologies (www.idtdna.com). *Spodoptera* sequences were amplified using primers derived from earlier characterization of the COI region (Nagoshi et al. 2006; Nagoshi et al. 2007), COI-*45F*, 5′-TTCGAGCTGAATTAGGRACYC -3′ (Y = C or T; R = A or G) and COI*-914R* (5′-GCWGATGTYAAATAWGCTCGWG -3′ (W = A or T) that are predicted to produce an 814 bp fragment from coordinate +101 to +914 (Figure 1). Overlapping sequence information was obtained using the same two primers for the internal 771 bp region that does not include the primer sequences.

### DNA sequence analysis.

The isolated fragments were analyzed by DNA sequencing performed by the University of Florida Interdisciplinary Center for Biotechnology Research using primers described for the PCR reactions. The quality of the sequence data was confirmed by examination of the chromatographs. In five cases involving *S. litura* samples, the chromatographs showed dual peaks of similar height at 10–20 nucleotide sites, suggesting that these samples are “heterozygotes” containing two COI sequences with high similarity that differed at the ambiguous sites. In contrast, 18 other *S. litura* samples gave the expected single sequence for the amplified COI region. The unambiguous sequences were assumed to be representative of the *S. litura* mitochondrial gene and used to derive the presumptive sequence of the “contaminant”. From the sequence data, two restriction enzyme site polymorphisms were identified, a Dra I site present only in the *S. litura* COI gene, and an Msp I site present only in the presumptive contaminant. To separate the two templates, the amplified PCR product from the heterozygotes was digested with one or the other restriction enzymes, and in each case the uncut fragment was gel-isolated and analyzed by DNA sequencing. The additional sequence carried in four of the five heterozygous specimens was identical to the *S. dolichos-1* haplotype, while the fifth displayed the *S. pulchella*-1 haplotype. The identity of the additional sequences with that of other specimens analyzed contemporaneously suggests cross-contamination, and these were not included in this study.

DNA comparisons and alignments were performed using the DS Gene program (Accelrys, www.accelrys.com) and the CLUSTAL algorithm. Descriptive DNA sequence statistics and calculations of nucleotide variation based on the Jukes-Cantor (JC) model were performed using DNAsp version 5.1 (Librado and Rozas 2009). Sequence divergences among individuals were calculated using the Kimura 2-Parameter distance model (Kimura 1980) and graphically displayed in a neighbor-joining (NJ) tree (Saitou and Nei 1987). Confidence was assessed by bootstrapping at 2000 replications with the *Bombyx mori COI* sequence (GenBank #EU141360) and *Helicoverpa armigera* COI sequence (GenBank #HQ132369) as outliers. All haplotypes obtained in this study have been deposited in GenBank: *S. frugiperda* (accession nos. HM136586–HM136602), other *Spodoptera* spp. (accession nos. HM756074–HM756093). Voucher specimens were deposited at CMAVE (Gainesville, FL).

## Results and Discussion

DNA sequences from a portion of the COI region were analyzed from five *Spodoptera* spp. native to Florida. These included the three major *Spodoptera* pests in the USA (*S.* *frugiperda* (rice-strain and corn-strain), *S. eridania,* and *S. exigua*), and two species attracted to *S. litura* pheromone traps, *S. dolichos* and *S. pulchella* (Meagher et al. 2008). These were compared to barcode sequences identified from two potentially invasive species, *S. littoralis* and *S. litura* (Table 1). Alignment of the COI sequences found no deletions or insertions and no stop codons, consistent with the amplified DNA arising from functional COI genes. Multiple haplotypes were found for all species except *S. littoralis*, with the highest haplotype diversity observed in *S. eridania* (Table 2). The majority of nucleotide substitutions were synonymous (25/27), with all but three mapping to the third codon position.

**Figure 2.**
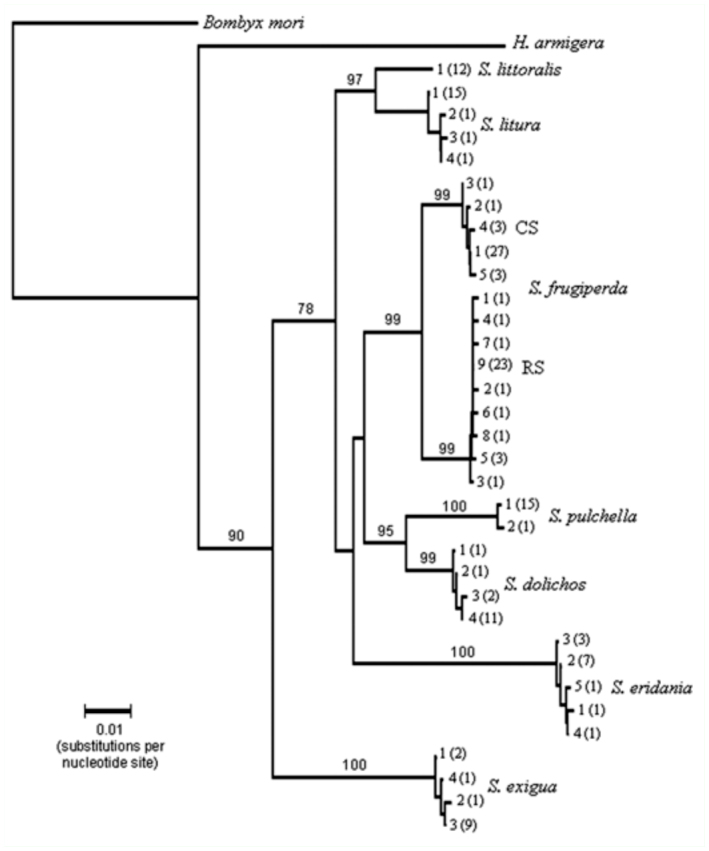
Neighbor-joining tree based on Kimura-2-Parameter distances for COI DNA sequences for different *Spodoptera* species. DNA sequences representative of the different haplotypes were used. Numbers next to species names identify haplotype category. Number of each haplotype found in parentheses. Numbers at branch points indicate 2000X bootstrap value. COI sequence from *Helicoverpa armigera* (accession # HQ132369) and *Bombyx mori* (accession #EU141360) were used as outlier sequences. High quality figures are available online.

The existence of a substantial barcode gap within this dataset was evident as the average nucleotide divergence within species was 0.08%, compared to an overall average divergence between species of 5.35% (Table 3). For each pairwise comparison, the mean variation between groups was substantially greater than the intra-group variation, surpassing the 10-fold threshold recommended for the barcode gap. Even the smallest divergence found between populations of 2.13%, observed between the two *S. frugiperda* strains, was 13-fold greater than the largest intraspecies divergence of 0.16% observed for *S. eridania.* This indicates that if the genetic variations exhibited by the small sampling groups are representative of the general populations, then COI sequence comparisons should be sufficiently sensitive to distinguish between *Spodoptera* species. This was confirmed by neighbor-joining phenetic analysis that differentiated at > 75% bootstrap values from the expected species, with *S. exigua* being most divergent (Figure 2). Closest similarities were found between *S. littoralis* and *S. litura*, *S. dolichos* and *S. pulchella*, and the two *S. frugiperda* host strains, but even these pairs segregated at > 95% bootstrap values.

These phenetic relationships were generally consistent with the phylogeny derived from morphological characteristics (Pogue 2002). Those cladistic studies identified *Spodoptera* as a monophyletic group with *S. exigua* as the most plesiomorphic species and *S. littoralis* and *S. litura* as closely related sister species. Discrimination of *S. littoralis* and *S. litura* is limited to comparisons of adult genital morphology (Mochida 1973; Ellis 2004). Therefore, the development of diagnostic barcodes will be of use for immature stages. Furthermore, it may be possible to discriminate between many *Spodoptera* spp. by sequence analysis of relatively short portions of the COI gene. An approximately 200 bp segment contains 36 polymorphisms that in combination readily differentiate between the *Spodoptera* groups tested in this study (Table 4, Figure 1). This means that even poorer quality specimens that allow only short PCR amplification products can potentially be used to at least delimit, if not completely specify, species identity.

These results support the feasibility of using DNA barcodes to rapidly assess the threat posed by an unknown specimen, either as a complement to morphological analysis or as the primary diagnostic indicator in cases where the requisite morphological keys are unavailable or compromised. This would entail comparing the unknown barcode sequence to barcode databases using pairwise sequence divergence calculations (e.g., the Kimura 2-parameter model) as visualized using a neighbor-joining tree—a methodology used effectively in a case study monitoring for invasive tussock moth species (Armstrong and Ball 2005; Ball and Armstrong 2006). If the unknown displays stronger barcode similarity to a quarantine species than native populations, it would be reason to recommend more extensive monitoring of the relevant areas. Currently, the major limitation of this approach for *Spodoptera* is the relatively small number of barcode sequences analyzed, both in terms of the range of species characterized and the number of sequences describing each group. The potential of insufficient sampling has challenged the validity of observed barcode gaps in other taxa (Wiemers and Fiedler 2007). Therefore, until more representative databases can be developed, species assignment using this approach should be considered tentative, pending confirmation by other methods. Nevertheless, the results described here suggest that the *Spodoptera* species complex can be readily differentiated by barcode comparisons and that even the preliminary barcode database from this study can indicate whether a particular specimen merits concern.

DNA barcoding makes possible the use of specimens at developmental stages where morphological keys for species identification are not available or of poor quality. In addition, continued advances in molecular genetic technology will improve the efficiency and economics of barcode analysis, making the screening of even a large number of samples increasingly practical. These benefits combined with the observed applicability of barcoding for species assignment in *Spodoptera* justify efforts to expand the barcoding database to become broader and more representative of the relevant domestic and exotic species. This could include non-*Spodoptera* species, such as members of the *Mythimna* and *Helicoverpa* species complexes, whose juvenile stages feed on many of the same hosts as *S. litura* and *S. littoralis*, and can be difficult to distinguish from *Spodoptera* by morphological criteria. As the barcoding database expands, so will the accuracy and utility of this approach for assigning species identity to unknown specimens, making it a valuable complement to the morphological methods currently used for the monitoring of invasive *Spodoptera* and other Lepidopteran pests in the United States.

**Table 1.**
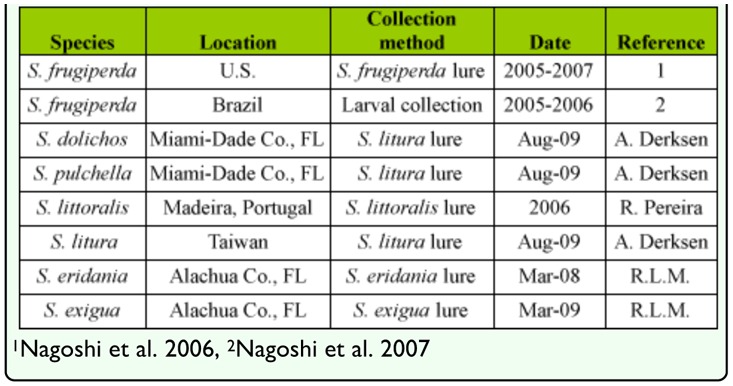
Source locality and host information.

**Table 2.**
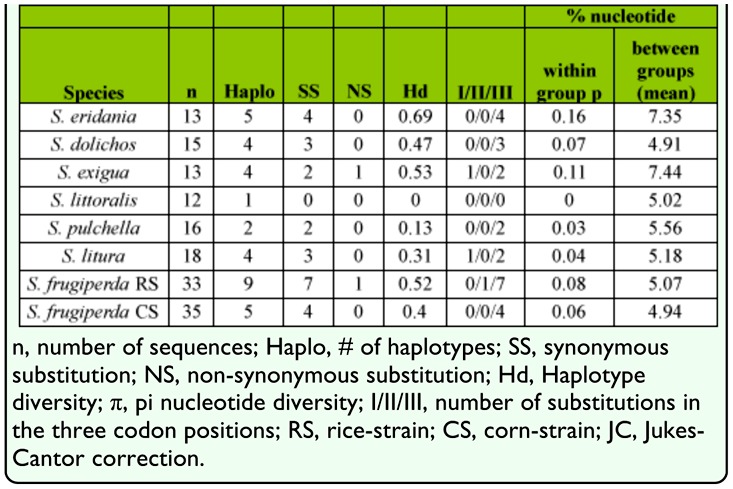
Descriptive statistics of polymorphisms found in a 771 bp portion of the *COI* gene from different *Spodoptera* species.

**Table 3.**
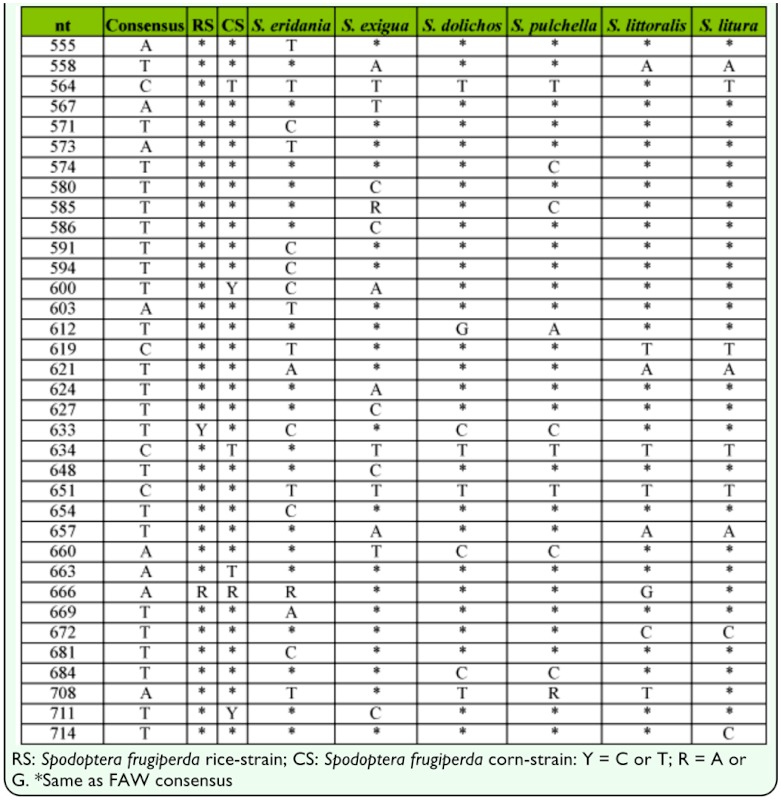
Diagnostic sequence polymorphisms in 160-bp segment of the COI gene.

## Abberviations:

**COI**,: cytochrome-c oxidase subunit
